# Australian women’s perspectives of routine enquiry into domestic violence before and after birth

**DOI:** 10.1186/s12884-023-05345-7

**Published:** 2023-01-19

**Authors:** Grace Branjerdporn, Tanya Clonan, Jennifer Boddy, Kerri Gillespie, Rosemary O’Malley, Kathleen Baird

**Affiliations:** 1 Gold Coast Hospital and Health Service, 1 Hospital Blvd, Southport, QLD 4215 Australia; 2 Griffith University, Southport, Australia; 3 Domestic Violence and Prevention Centre, Southport, Australia; 4 University of Technology Sydney, Sydney, Australia

**Keywords:** Domestic and family violence, Women, peripartum, Maternity services, Trauma-informed

## Abstract

**Background:**

Peripartum women are vulnerable to experiencing intimate partner violence (IPV). Interactions with health practitioners during maternity care provide a unique opportunity to detect and respond to women who are experiencing IPV. The aim of this study was to explore women’s experiences of IPV screening at an Australian maternity service.

**Methods:**

Qualitative methodology was used in this cross-sectional study. In-depth semi-structured interviews were conducted with women with IPV who attended an Australian maternity service. Thematic analysis was used to identify codes and themes.

**Results:**

The nine women expressed three major themes, and six sub-themes, surrounding clinician approaches (communication and support, asking about IPV, and following disclosure), system considerations (fear of child safety involvement, continuity of care, and environmental considerations), and education. All participants supported screening and highlighted beneficial or detrimental approaches to screening and care, and recommendations for improvement.

**Conclusion:**

This research points to the benefit of trauma-informed frameworks in hospitals to support women experiencing IPV.

## Background

Domestic and Family Violence is a complex social problem, disproportionately affecting women and children [1]. Intimate partner violence (IPV) is one of the most common forms of domestic violence, and describes any form of violence, abuse, or controlling behaviour within an intimate relationship [2]. The World Health Organization (WHO) [2] estimates that around 30% of women will be subjected to physical and/or sexual violence in their lifetime, predominantly by an intimate partner. The presence of children within relationships is known to increase the likelihood of violence against women; with mothers and pregnant women three times more likely to experience IPV than women without children [3]. According to the WHO [4], IPV homicide is the leading cause of death for pregnant women globally. Previous studies [5, 6] suggest IPV affects 2–3% of women during pregnancy in Australia. Data collated by the Australian Bureau of Statistics (ABS) observed that 18% of women who experienced physical IPV with a current partner continued to experience physical violence during pregnancy; for women who reported having experienced IPV with a previous partner, 48% reported that the first incidence of violence commenced during their pregnancy [7].

The effects of IPV on women and children, particularly during pregnancy, are complex; ranging from physical to mental health impairments, which can lead to harmful maternal behaviours that can significantly compromise the health of the foetus and affect parenting abilities [3, 4]. The risk of violence during pregnancy is greater in women aged 18 to 24 years [8]. The adverse maternal health impairments associated with IPV range from acute injury to adverse chronic health outcomes and include recurrent miscarriage, physical injuries, mental health manifestations (depression, post-traumatic stress disorder, anxiety), chronic pain, and unexplained gastrointestinal symptoms [9]. Harmful maternal behaviours include nutritional deficiency; consumption of drugs; prescribed and illegal, alcohol and nicotine, and delayed pregnancy care [4, 9, 10]. Almost 67% of children who are exposed to domestic violence are likely to experience a range of developmental and adjustment difficulties related to vicarious trauma and impaired attachment [11, 12, 13].

Interactions with midwives and other health care practitioners during maternity care provide a unique opportunity to detect and respond to women who are experiencing IPV. The WHO [14] has released guidelines on the appropriate treatment of IPV that addresses women-centred care, staff approaches, routine screening, and appropriate care, interventions, and referrals. Many of these recommendations have been incorporated into DV training [12] and education programs designed for clinicians [15, 16]. Unfortunately, previous concerted efforts around routine enquiry in maternity services and clinician education appear to have had a limited impact upon women’s willingness to disclose IPV or accept referrals for IPV support services [17]. While previous studies have found that the majority of women find routine IPV screening acceptable and beneficial, a number of organisational and systemic barriers to IPV screening and identification within health services have been identified [18]. The main barriers reported have been a lack of organisational resources to support women who disclose IPV; clinician knowledge, training, and confidence to screen; and the attitudes of clinicians regarding IPV [19, 20].

Screening and identification rates remain low despite more attention and funding being directed toward IPV education, detection, and support services [10, 21]. For this reason, regular and up-to-date research to examine and evaluate the processes, barriers, and enablers to the identification and response to IPV is critical. This study was designed to deepen our understanding of how women experiencing IPV interact with maternity care services in a large public tertiary hospital in Queensland, Australia, and the factors that influence their choices around disclosure and help-seeking. The study aimed to develop an understanding of which aspects the women found to be conducive to engagement as well as identify any elements of the practice that acted as barriers to help-seeking. The study has drawn upon Liang’s [22] theoretical framework for understanding help-seeking processes in IPV. This framework builds upon the transtheoretical model of behaviour change and incorporates the roles of social, cultural and environmental influences on women’s transition through the stages of problem recognition, decision to seek support, and the selection of a support provider. Liang [22] identifies that women’s conceptualisation of IPV and her help-seeking behaviour are bi-directionally influential factors. The framework highlights the importance of exploring the negative supports experienced by women (described here as barriers to disclosure) and the decision-making processes around support-seeking and support selection.

## Methodology

Qualitative methodology was used in this cross-sectional study. Individual interviews were conducted and analysed thematically. The framework by Braun and Clarke [23] informed the selection of a contextualist epistemological approach to thematic analysis for its recognition of the experiences as well as meanings and lived reality of the participants. This framework presents a guide of six phases that can be used in a non-linear and recursive fashion: (1) familiarisation of data; (2) data coding; (3) theme searching; (4); theme reviewing; (5) themes refinement and definition; (6) interpretation and write-up. The Braun and Clark framework was chosen for its systematic yet flexible approach that ensures reflexivity and interpretive rigour [24].

### Ethical considerations

Approval for this study was obtained from the relevant Human Research Ethics Committee. All participants provided written and verbal consent to participate. Where possible, the researchers aimed to mitigate any risks to participants due to the sensitive nature of the research. Participants were interviewed by a researcher of the same gender as the participant. Interviews were held in a private room within a local domestic violence community centre that the women regularly visited. The centre was attended by domestic violence community support workers known to the women, who were available for support should the interviews cause distress or discomfort. The research team also obtained free childcare for the women on the day of the interview. Participants were offered a $30 retail voucher to compensate for their time and travel expenses. Pseudonyms have been used throughout the paper, and all identifying information has been withheld from the manuscript to ensure the privacy of the participants.

### Sampling and recruitment

A convenience sampling method was used to recruit women who had attended a large tertiary hospital maternity service within the past five years, who had also been identified as experiencing IPV. Women were identified by domestic violence community support workers and clinicians at a community-based support service, women’s playgroups for vulnerable mothers, and mental health support within a psychiatry and nursing team. Community support workers and clinicians at these facilities gave eligible participants invitation fliers for the study. Interested women then contacted researchers by email or phone to organise further information, consent, and to schedule interviews. Each participant had at least one experience of IPV, was engaged with a domestic violence or mental health support agency at the time of their interview, and had received perinatal maternity care at the hospital. Targeted recruitment was conducted over an 18-month period. While a repetition of responses was observed toward the end of data collection indicating saturation, recruitment of women for this study was challenging. This was likely due to barriers such as homelessness, privacy concerns or fear of stigma (particularly if they are still cohabitating with their perpetrator), and the discomfort of discussing their experiences with researchers (Dichter et al., 2019 [25]).

### Data collection

A mental health occupational therapist, trained in qualitative methodology and independent to the maternity care, conducted in-depth, semi-structured interviews (*N* = 9). The primary questions asked the women to described their experience of being screened for IPV during pregnancy; what they considered were the helpful and unhelpful aspects of the midwives’ approach in discussing IPV; and what recommendations they had for the health service around the screening process. The interviews were conducted face–to–face in a private room or via telephone to optimise the safety and comfort of the participant. The average interview duration was 60 min. Transcripts were digitally audio-recorded and transcribed verbatim in a non-identifiable format.

### Data analysis

After an initial reading of each interview, transcripts were analysed thematically using NVivo 12 Pro. An inductive approach to the initial coding was undertaken by (TC); an undergraduate social work honours student, to ensure sensitivity to the meaning and context of the data, as described by Skjott Linneberg and Korsgaard [26]. Iterative coding was used until themes were developed and assessed by independent researchers, members of the research team and co- authors.

## Results

Overall, 9 women participated in the study (see Table 1 for details). The women had a mean age of 30.7 years, an average of 2 children each, and 2 participants were pregnant at the time of their interview. All women were heterosexual and Caucasian. The study was predominantly interested in how women interacted with midwives around routine enquiry; however, women also discussed their interactions with obstetricians, social workers and student midwives. The term clinician therefore is inclusive of all health professionals listed, except where specified. Analysis of the interviews uncovered three major themes: clinician approaches; system considerations; and education. Each major theme may also contain multiple sub-themes to provide a clear and comprehensive explanation of the study findings.

**Table 1:** Participant Demographics (*N* = 9)

Pseudonym	Relationship Status	Number of Children
Jessica	Separated	4
Anabel	Separated	1
Chelsea	Separated	1 + pregnant
Joanne	Unclear	1
Rachel	Unclear	3
Stella	Separated	2 + pregnant
Sophie	Married	2
Linda	Separated	2
Sally	Married	2

### Clinician approaches

Women’s perceptions of the methods and manner in which clinicians approached and responded to IPV throughout maternity care are reported according to three sub-themes: *communication and support*; *asking about IPV*; *post-disclosure*.

#### Communication and support

In general, women were empowered and felt safe to discuss their IPV experiences when midwives took the time to develop a trusting relationship which was based on compassion and respect:

“Just bring it up and just be like, are you feeling comfortable with being at home; are you safe? But say it in a nicer way, like they should say that to every woman that comes in” (Linda).

“Just that they’re open with me. If I’m honest and open with you, you’ve got to be honest and open with me. Like again, two-way street” (Chelsea).

Conversely, highly clinical interactions and a sense of being judged based on the father was described as the antithesis of making women feel safe to discuss IPV:

“. . maybe not just flick through pages so fast and talk to us about the questions would be good, because they’re kind of filling everything out, but you don’t actually know what they’re writing down. I felt that was quite stressful and a bit rude. . .” (Sophie).

One woman was concerned about how professionals would react to her disclosure about IPV when she had a history of substance abuse. She was concerned that they would prejudge her based on her earlier history:

“I stopped when I fell pregnant and I’ve been clean ever since. But they would just be very judgemental. They were judging me because of him. I wasn’t like him” (Chelsea).

Women also made specific comments about particular professional disciplines including *midwives*, *obstetricians* and *social workers*.

Characteristics women appreciated of *midwives* were when interactions were “warm” (Jessica), “supportive” (Sophie), and “there for us” for both baby and mother (Joanne). Negative attributes were when midwives “seemed really rushed” (Sophie), were not treated “on a human level” (Rachel), and/or were treated unfavourably based on the husband: “I also felt like I was being penalised for the behaviour of my husband, who I was the victim of” (Jessica).

*Obstetricians* were generally perceived to focus on biomedical rather than social matters. “Sometimes you see the obstetricians and they’re great, but they are a little bit removed from the social stuff” (Jessica). It was welcomed, however, when obstetricians took more time to build rapport, understand the biopsychosocial issues, and make appropriate IPV referrals: “. . I had one obstetrician which was very kind. . and had that nice blend between the more holistic approach that some of the midwives have and that medical expertise…You’d go to an appointment and some obstetricians would say, yes, I’m on to it and do it straight away and that would happen. Others would say, we’ll get that happening and nothing happened and everything would get forgotten about” (Jessica).

Finally, women considered *social workers* to be IPV specialists, particularly in listening and assisting with completing forms, while also connecting the women with appropriate hospital and community services: “I think the most important thing is to feel safe, then you need somebody who wants to listen, obviously someone who is educated in domestic violence and they [social workers] know how to counsel you on it and all the other avenues” (Anabel).

#### Asking about IPV

All the participants in the study accepted routine enquiry as necessary and beneficial; including those who did not recall being asked themselves: “I think that would have been quite helpful” (Angela); “. . ask about how. . what’s going on, or ask about the past, whatever. Get some brief, a background of who you are and all that sort of stuff and then go from there maybe (Chelsea); and “… they’re taking extra measure to make sure that nothing bad is going on at home, which was good” (Rachel).

The majority of women suggested that clinicians should learn to notice signs of IPV in the behaviour of women and their partners and to be confident in taking appropriate action. “Once they spoke to me, they saw that it was his manipulation. So, I think they had some kind of insight into his behaviours” (Jessica).

Privacy was considered an essential element, including a closed door, and creating a reason for partners to leave the room should they be in attendance. One suggestion was to dedicate one antenatal appointment to “women’s business”, excluding partners altogether; while the majority favoured midwives taking a flexible and “creative” approach to finding time alone with the woman, such as body examinations and urine sample tests, to enable private personal conversations:

“. . you still have to feel like you’re in a safe environment and that he’s not going to find out or he’s not going to hear. .” (Annabel).

One woman was concerned that asking the partner to leave was not a good idea and indicated that her partner would insist that she disclose everything that had been discussed in his absence stating:

“He would want to know everything…. it wouldn’t have ended until I told him exactly what was said, in the room when he was not there. . this situation would be avoided. . if they didn’t ask him to leave.” (Rachel).

The women suggested that clinicians take a “gentle” (Anabel), “friendly” (Stella), “sensitive” (Sophie), and” conversational” (Stella) approach to enquiry. They recommended using broad open questions; and then taking cues from the woman’s responses to ask more specific questions about her physical, emotional, financial and psychological safety:

“Then someone would be able to pick up on where to go from there. . It would signify whether they could pursue that conversation or not” (Rachel). Anabel similarly reported, “… how are things at home in your relationship, are you feeling like … you’re going to get enough support, that you feel safe?”. Sally, on the other hand, experienced a confrontational approach which was not preferred:

“I had a black-eye and so she shut the curtain and she kind of got a little bit in my face…she got quite close and said… “You want to tell me what’s going on?” I could understand her concern, but it was just a little bit too close… it wasn’t kind of eased into…it was kind of, you know, “Do you want to tell me what’s going on?” and I’m sat there thinking “Not really”. At the same time, I was thinking, you know I wanted to tell someone because I was in…I mean I burst into tears telling her because yeah, I just again couldn’t kind of comprehend like where my life was at this point.

Some women suggested a written screening tool or watching a video before or during the appointment was considered a useful approach to starting the conversation and preferable to asking the questions in a verbal format:

“It is a lot easier because you wrote it down and they can ask you questions regarding exactly what you wrote” (Sophie).

The women also suggested repeated routine IPV screening at appointments throughout the antenatal, intrapartum and postpartum care phases would be helpful:

“… I think it’s still good if you can say something because you’re right; the first time people might be uncomfortable saying anything and then if they see them again the second time - I think it’s still quite good to just say, how’s everything at home, how’s your husband coping with everything, because that may get people talking as well. So yeah, I think that could be quite helpful actually, asking them over a few sessions or whatever.” (Anabel).

#### Following disclosure

The women provided examples of both positive and negative interactions with midwives in appointments subsequent to IPV disclosure, and discussed communication with, and between midwives involved in their care. Linda appreciated the way clinicians responded to IPV history on their files: “. . they would look at my record and see that I’ve, you know, had domestic violence, and they ask if everything’s all right. . they were asking are we safe and all that stuff so, that’s good”.

Conversely, Stella described how she felt when a midwife addressed the IPV note in her file in an abrupt manner: “…. it felt like it was a putdown. Like. . Oh, you’re in a domestic violence - you’re still talking to the father…it felt judgemental”.

In situations where the partner was present at the hospital post-disclosure, the women expressed a desire for midwives to intervene in a calm, confident and non-confrontational manner. Anabel explained, “She had interaction with him as well. There was a bit of a scene where he came and confronted me, and she really was an advocate for me, that was really good”.

Stella recounted another successful approach of a midwife whom she felt advocated for her: “She was [empowering] - because she was the one that was making herself in control. She was the one that she knew what she was doing, that was her job but she was trying to distract him in his certain way. Like, oh no, we - yeah, we’ve got to go - we’ve got to check up on her urine test, or something like that.”

One woman who had disclosed IPV suggested it would be good to contact particular midwives between visits: “Yeah, that will also be good. Have them like, for instance, a personal mobile number that you could even swing a text to, like hey, is this right? Just something like that” (Chelsea). Another participant requested that refuge support be available at the hospital as suggested by a midwife:

“She had seen me directly after the assault. She was lovely and she said to me if you don’t feel safe then just call the hospital and you can bring the babies with you, come in for another night. She said, there are cots everywhere. We’ll be able to make it work. Then that situation came up, and when I called the hospital, they were, like, what? What are you talking about? We don’t do that. I was, like, oh, okay. I think maybe having that consistency so that you know there’s some way that you can - that’s down in notes or something. . .” (Jessica).

### System considerations

Women reported three sub-themes related to the organisational and hospital context, including *fear of child safety involvement*, *midwifery continuity of care*, *and environmental considerations*.

#### Fear of child safety involvement

The women weighed up the potential benefits of discussing IPV against the perceived threat of involuntary intervention by child safety services, with one woman having personal experience of this happening to her:

“I think often it’s really hard to disclose stuff that’s going on if you feel that then you’re going to be put on the radar…. You feel like, I don’t want to tell you these things because I feel like I’m putting myself at risk, my parenting is being judged and you’re not actually going to do anything to help me”. (Jessica)

“So, then she [the midwife] said “Look, you do realise now I’m gonna have to report this.” Which I didn’t…then we’ve gone from, you know, all this stuff to then having the police and child services rock up on my door, umm but yeah, it was, it was so confronting” (Sally).

#### Midwifery continuity of care

Women experienced three different models of midwifery care: Shared Care (i.e., various midwives and doctors including General Practitioners are seen throughout the woman’s pregnancy), Midwifery Continuity of Care (MCoC) (i.e., a known midwife provides care throughout the woman’s pregnancy, birth and postnatal period), and Midwifery Student Continuity of Care (MSCoC) (i.e., a student midwife accompanies a woman throughout her pregnancy under the guidance and accountability of a qualified midwife) (Australian College of Midwives, 2020 [27]). Overall, the women preferred to receive maternity care via continuity of care models as women who experienced shared care explained:

. . you spend a lot of your visit rehashing your history. . it is traumatic. Some of it is perhaps embarrassing. . I have very few criticisms of the hospital except from that. I understand it’s hard to manage, but the lack of consistency, particularly when there are a few health issues and a few social issues - it’s quite hard when you see someone different each time. (Jessica)

Rachel reported that MCoC “. . would have been great because. . they know your story. . they’re familiar with who you are, not always building a relationship each time”. While Sophie preferred the dedicated MCoC, she was highly appreciative of the student midwife’s support and advocacy:

. . when you’re not seeing the same midwife in your appointments. That was a bit annoying, but it was good that I had the student midwife, seeing the same person every time and knows you. . She can kind of helps me if I get stuck, which was really good. (Sophie)

#### Environmental considerations

Several women discussed modifying the environment to support women to feel comfortable and safe to disclose. Stella and Sally also suggested creating a women’s only “safe space” whereby women could be safe from their partner attempting to visit, feel relaxed, and access IPV information and support: “Even just having that safe space in the maternity ward. . you can go out and just have a tea and maybe talk to the other mothers that might be going through it, you know?. . that’s where you could probably build a group, from there even and just go - transition to DV counselling or DV. . courses.” (Stella).

“I think there needs to be a bit more of a safe space for people to be able to kind of express these things or you know, kind of be given more information in more of a kind of structured but relaxed way… when I spoke to that midwife who detected you know “I got a black eye” and “Here we go”…it perhaps would have been nicer to go to you know, somewhere that had a lounge or whatever to you know kind of feel a bit more homely and bit more safe and relaxed” (Sally).

### Education

The third main theme to emerge from the data analysis was related to education. The women provided commentary on the importance of educating people about IPV, including when to provide education, possible topics in understanding IPV, and ways of delivering the education through peer support and other various mediums. Three women commented about the inpatient postpartum phase as a unique opportunity for education through brochures and videos and sensitive conversations away from partners: “It [time after birth] gives an opportunity for a midwife or a doctor to ask that question…” (Linda). : “. . just allowing that opportunity to have more time in hospital so that. . supports can be put in place for after they’re discharged…that [video about IPV] could be an idea as well, because the person’s [the partner is] often not there, so gives you a little bit of space. . you put that little bit of grace there, that time of grace where if you want to, you can just watch it …(Rachel).

This cohort of women strongly supported society-wide public education to increase awareness about what constitutes IPV, particularly focusing on coercive control and patterns of non-physical abuse. As explained by Rachel: “… just some way to help women understand, and it’s not just physical… Yeah and even like a little, I don’t know, PowerPoint presentation of red flags, how do I identify, you know is this happening to you”; and expanded upon by Anabel: “It’s emotional, it’s psychological, it’s sexual, it’s control, it’s power and all that, which was a big eye opener for me”.

Women were enthusiastic about the potential of peer support and education in the form of online forums, face-to-face groups, videos and written media for its ability to provide a depth of understanding, hope for the future and confidence in the system that helped women before them: “Someone who has been in the situation. . There is hope. A lot of them don’t think that there is and they can’t get out of it, but there is” (Chelsea).

A common view amongst participants was that educational resources such as pamphlets, brochures, information cards, booklets and posters should be widely available in maternity waiting rooms and toilet facilities: “. . even when you’re drying your hands at the toilet,. . a sign that says, are you experiencing any of this, call DV Connect or blah, blah, blah. Just that little thing while you’re standing there.” (Anabel).

Additionally, selected information should be provided to all women as routine inclusions in hospital provided maternity information and sample packs: “. . getting some brochures or pamphlets popped in there because you read everything. . these are the signs and the symptoms” (Anabel); and “Chuck on in there, so it’s less obvious. . .so it’s not that invasive and obvious” (Stella).

## Discussion

The women in this study shared their perceptions of clinicians’ approaches to IPV, and which approaches they believed would encourage more women to disclose and accept help for IPV during maternity care. The literature establishes that feeling cared for, validated and supported by the clinician is one of the essential elements for women to engage with discussions of IPV [17, 18, 28, 29, 30, 31]. Overall, women feel safer discussing sensitive social issues like IPV when clinicians use a friendly and open conversational communication style that conveys a non-judgemental and genuine interest in their welfare as a whole person, human to human. This interpersonal approach normalises the discussion of IPV, and it is helpful for women who are struggling with feelings of shame, self-blame and fear that the system response will worsen their situation. Assuming that clinicians adopt the appropriate relational communication approach, women support the practice of routine enquiry and suggest that it should be repeated at appointments throughout the antenatal, intrapartum and postpartum care phases.

Revisiting the topic and question of IPV through repeated routine enquiry creates multiple opportunities to educate childbearing women about the presentation of IPV and allows for the incremental shifts in their understanding and the dynamic nature of family relationships over time. Completing a brief questionnaire, watching a video, or being given an information card before being asked about IPV was considered helpful, both as an educational tool and an ice-breaker to help the women feel safe to talk about IPV.

The repetition of routine enquiry also serves to normalise the discussion of this sensitive topic which allows women to trust that there is help available if they needed it. Previous studies support the findings that women see routine enquiry as necessary and consider it to be beneficial as an intervention in its own right, regardless of whether a woman is experiencing IPV at the time or not; and whether she chooses to disclose at that time [17, 18, 19, 28, 30, 32, 33]. Because disclosure of IPV is a process that occurs over time [17, 33], the majority of women encourage repeated routine enquiry throughout antenatal and postnatal care to allow for the dynamic nature of family relationships [19, 28, 29, 33].

The current study demonstrates a strong understanding of how women perceive maternity care responses to IPV, however translating that into practice continues to be a challenge for large healthcare institutions. The method for overcoming the barriers to disclosure falls disproportionately on the interpersonal skills of midwives, yet many of the barriers remain systemic. Women’s support for the benefits of routine enquiry regardless of disclosure calls into question the common practice of using disclosure and referral rates to evaluate the benefit of routine enquiry and encourages a fresh review of the real goals and success measurements of routine enquiry. The well-established datum that women find benefit in routine enquiry regardless of disclosure suggests that disclosure rates are not an accurate reflection of positive impact, and the individualised nature of the journey that women take through IPV could be better accounted for by reviewing the objectives of various interventions.

Further to supporting the practice of repeated routine enquiry, the women identified the inpatient postpartum phase as a unique opportunity for education and a sensitive discussion about IPV because they had time away from their partners. Another unique recommendation of this study was to create a mother’s only space that is warm, comfortable, welcoming, safe and secure. The women envisaged the space as being staffed by a IPV clinician and used for women to connect with other women, watch educational videos, get information, and access crisis counselling and referrals.

The majority of the women’s contact was with midwives who are generally perceived to be warm, helpful and open to discussing social issues. Women prefer a Midwifery Continuity of Care (MCoC) model because it allows them to build upon the relationship on each consecutive visit, follow up on matters raised in the previous visit and make plans for future appointments. In the absence of a MCoC service model, a student-midwife continuity of care model provided a similar woman-centred experience of someone to accompany and advocate for them across the maternity journey. Upon disclosure of IPV, women valued written referral and personal introduction to a IPV specialist for counselling and practical support.

The women emphasised IPV education in their journey to safety and consistently contextualised their responses according to their situation and understanding of IPV at a specific point in that journey. The framing of the women’s responses suggested three distinctive phases for the types of information and support the women found helpful: 1. understanding what behaviours and actions constitute IPV (understanding); 2. realising that their partner is perpetrating IPV (realisation) and; 3. becoming ready to enact change and accept help (readiness). The women supported the availability of educational resources such as pamphlets, brochures, information cards, booklets and posters amongst the various other health promotion materials. Centring the process of understanding, realisation, and readiness can inform the development and delivery of tailored education, resources and support to meet the needs of women as they journey towards safety.

The current study confirmed the most common barriers to disclosure across the literature. Fear that the consequences of disclosing and seeking support for IPV would worsen their circumstances remains the most common barrier to help-seeking. Women reported being afraid that if their partner were to find out about their disclosure, their existing safety strategies would be compromised, and their partner may retaliate [17, 29, 30, 31, 33]. Women also reported hesitancy to disclose IPV for fear that it would trigger the involvement and potentially punitive actions of child safety and police departments as midwives were obligated under Child Protection Act 1999 to report any child protection concerns [17, 19, 29, 30, 33, 34, 35]. The women in the current study were very clear that they were hesitant to open themselves up to scrutiny and criticism until they were certain that they would retain their autonomy, along with support to exercise it. Women also felt judged by midwives when they did not leave the abusive relationship, which points to the lack of awareness of the complexities and risks for leaving a relationship. Women also lacked the confidence that help was available or would change anything, indicative of the isolation and the sense of hopelessness they felt.

In conclusion, the knowledge of what women consider helpful and their perceptions and preferences for maternity service care is available, but it lacks coherence and the mechanisms required for the successful translation of research into practice. A practice framework that recognises and responds appropriately to victims of trauma and violence is needed to support the midwives and ensure ongoing input from service users. It is recommended that further research is undertaken to (i) evaluate educational programs for women around IPV during pregnancy, (ii) develop suitable measurements for evaluating the impact of routine enquiry on disclosure rates and service access, and (iii) contribute to the development of trauma informed practice frameworks to support women experiencing IPV during pregnancy. In doing so, this will support the increased detection and response to IPV to support women and children.

### Limitations

The study had some major limitations relating to the size and representativeness of the sample. Due to the sensitive nature of the study, which included a number of safety and privacy concerns for the participants and researchers involved, only a small number of participants were able to be recruited. The study also was able to recruit only Caucasian and heterosexual women. The study also lacked interpreter support, further limiting the number of women eligible for inclusion. Socially and culturally diverse populations are known to possess their own unique issues and perceptions in terms of IPV screening experiences, approaches, and exposure to stigma and discrimination. The study therefore failed to capture many of the individual considerations relating to the screening and treatment of ethnically diverse and LGBTQ + communities. Future studies aimed at these groups would be beneficial to the effective detection and response of IPV in more diverse populations that make up a large portion of the current Australian population matrix.

## Data Availability

Data is not publicly available due to ethical and privacy concerns. Full, de-identified transcripts may be requested from the corresponding author (GB) on reasonable request.
